# *Vibrio* Species in Wastewater Final Effluents and Receiving Watershed in South Africa: Implications for Public Health

**DOI:** 10.3390/ijerph15061266

**Published:** 2018-06-15

**Authors:** Allisen N. Okeyo, Nolonwabo Nontongana, Taiwo O. Fadare, Anthony I. Okoh

**Affiliations:** 1 SAMRC Microbial Water Quality Monitoring Centre, University of Fort Hare, Alice 5700, South Africa; n.nontongana@gmail.com (N.N.); tosinfadare@yahoo.com (T.O.F.); aokoh@ufh.ac.za (A.I.O.); 2 Applied and Environmental Microbiology Research Group (AEMREG), Department of Biochemistry and Microbiology, University of Fort Hare, Alice 5700, South Africa; 3 Department of Biochemistry and Microbiology, University of Fort Hare, P/Bag X1314, Eastern Cape, Alice 5700, South Africa

**Keywords:** wastewater effluent, *Vibrio* pathogens, wastewater monitoring, public health

## Abstract

Wastewater treatment facilities in South Africa are obliged to make provision for wastewater effluent quality management, with the aim of securing the integrity of the surrounding watersheds and environments. The Department of Water Affairs has documented regulatory parameters that have, over the years, served as a guideline for quality monitoring/management purposes. However, these guidelines have not been regularly updated and this may have contributed to some of the water quality anomalies. Studies have shown that promoting the monitoring of the current routinely monitored parameters (both microbial and physicochemical) may not be sufficient. Organisms causing illnesses or even outbreaks, such as *Vibrio* pathogens with their characteristic environmental resilience, are not included in the guidelines. In South Africa, studies that have been conducted on the occurrence of *Vibrio* pathogens in domestic and wastewater effluent have made it apparent that these pathogens should also be monitored. The importance of effective wastewater management as one of the key aspects towards protecting surrounding environments and receiving watersheds, as well as protecting public health, is highlighted in this review. Emphasis on the significance of the *Vibrio* pathogen in wastewater is a particular focus.

## 1. Introduction

Water is a critical element and an important core for sustainable socio-economic development, along with the eradication of poverty and health discrepancies. The need for safe drinking water and proper sanitation management is a perennial concern for sustainable life globally [1]. Despite significant progress that has been made in this regard, discrepancies still exist. Globally, there are approximately 2.1 billion people who lack access to quality drinking water sources, and 4.5 billion that lack proper sanitation [2]. There are significant differences between more affluent and poorer communities, particularly in the developing world. This crisis is further exacerbated by increasing poverty, rapid urbanization and population growth as well as climate change, which can further stress an often-deteriorating water and sanitation infrastructure, putting many at risk of water and sanitation related discrepancies [3,4].

Proper management of a water treatment facility is pivotal to bettering the quality of water consumed (drinking water) or released (wastewater) into the environment [5]. In conjunction with the many determinants used to indicate the holistic health or quality of an area, wastewater treatment systems may suffice as a positive indicator of development [6]. The release of poorly treated wastewater into nearby watersheds directly threatens the macro and micro flora and fauna present; retarding the provision of good quality water required for societal functions [3].

Many wastewater treatment plants in South Africa still release final effluent containing significant amounts of enteric pathogens such as the *Vibrio* genus, known for its environmental resilience and relation to disease outbreaks. This results in the impairment of the surrounding receiving water bodies [5,7,8]. Wastewater facilities are obliged to make provision for quality management of their wastewater effluents prior to release into surrounding water bodies. There are microbial and physicochemical parameters which are required to be routinely monitored, indicated in the water quality guidelines; these have been the basis of water quality management and research over the years. However, evidence suggests that the organisms causing illnesses and outbreaks are not necessarily the ones which are routinely monitored. Studies have already indicated the need to monitor not only the classical pollution indicators, i.e., culturable total or faecal coliforms, but also viral pathogens, toxigenic *E. coli*, and highly infectious bacterial pathogens such as *Vibrio*. [5,8,9,10,11].

This paper focuses on sustainable wastewater management as a key approach towards protecting receiving watersheds and ultimately public health. Moreover, it highlights the significance of the *Vibrio* pathogen in wastewater, emphasizing their impact on public health and justifying the need to monitor for this pathogen.

## 2. Wastewater Complexities

According to Corcoran [12], wastewater is defined as a combination of one or more of the following: (1) domestic effluent (blackwater and greywater comprising of excreta, urine, faecal sludge, bathing and kitchen wastewater); (2) water from institutions such as hospitals, industries and other commercial establishments; (3) storm water and other types of urban run-off; (4) dissolved or suspended agricultural, horticultural and aquaculture waste. The composition of wastewater in its entirety is dependent primarily on its source, characterized by both its physicochemical content (temperature, turbidity, pH, colour, odour, suspended solids, total dissolved solids, dissolved oxygen (DO), biological oxygen demand (BOD), nutrients and toxic substances, organics, alkalinity, metals and chlorides) and biological or microbial content (plants and animals, bacteria, protozoa, helminths, viruses) [3,13,14].

The degree of environmental trauma of wastewater is directly related to its composition. The diversity of wastewater is brought about by factors affecting it, e.g., the natural environment, population, and/or the surrounding recreational, domestic and industrial related activities [13]. Municipal wastewater, for instance, is a combination of different inflows which include human excrement (sewage), suspended solids, debris and an array of chemicals originating from residential channels, industrial and commercial activities [15]; Table 1 shows some wastewater related contaminants. These factors also subsequently affect the discharge patterns of the wastewater and could impact on the chemical and microbial status of treated wastewater final effluent, in turn impacting on the surrounding water bodies [3,16].

### 2.1. Wastewater Treatment

**Figure 1:**
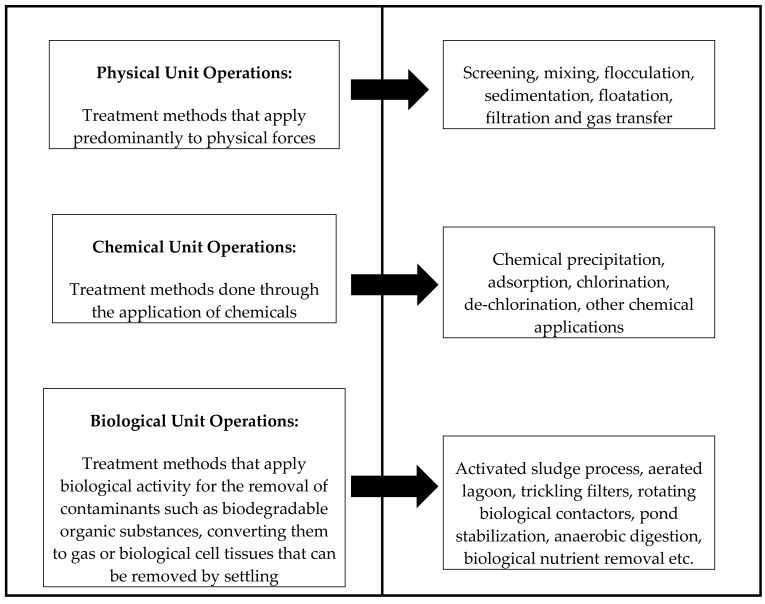
Summary of wastewater treatment unit operations. Source: [18,21].

The implementation of a proper treatment plant design or the selection of effective treatment technologies depends on the nature of the wastewater to be treated [18]. Wastewater treatment was conceptualised with the aim to enable the disposal of wastewater safely, without this water polluting the receiving water bodies, being a danger to public health, or causing other water related anomalies [19]. Attempted first in the 1900s, wastewater treatment is now implemented worldwide and is continually being improved on. Globally, the treatment of wastewater is considered paramount. It is one of the key aspects for water quality management and is deemed to provide a genuine solution to two major challenges: (1) protecting water resources, and (2) access to sustainable water and sanitation services [18,20]. Typically, wastewater treatment processes involve the use of physical, chemical and biological unit operations. A summary of the operations employed is illustrated below in Figure 1:

Expanding on the above unit operations, wastewater treatment undergoes the following fundamental stages: preliminary, primary, secondary and tertiary stages [22].

#### 2.1.1. Preliminary Treatment Process

Wastewater entering a treatment plant typically goes through preliminary treatment as the first process of treatment. The removal of larger debris such as paper, plastic and other large floating objects, for example, grit, and silt is performed at this stage. This stage prevents the accumulation of debris that may damage or clog the pumps, small pipes, other equipment and downstream processes in the treatment plant, is done at this stage. The screens are constructed from steel or iron bars and vary from coarse, with openings of about half an inch, to fine, generally mesh screens with much smaller openings. Materials removed by the screens are often dangerous and are safely disposed of using appropriate methods to a particular plant [6,23].

#### 2.1.2. Primary Treatment Process

The primary treatment process is comprised predominantly of primary settling and sedimentation. At this stage, wastewater still contains dissolved organic and inorganic components, as well as some suspended solids (i.e. sand, grease, fats, oils and grit). The removal of these components at this stage is key to avoid excessive amounts of large solids from interfering with other processes [23]. Wastewater at this stage enters sedimentation tanks which reduce the flow rate. The flow of the wastewater becomes almost stagnant and is held in the tank for several hours. The formation of primary sludge is apparent at this stage, with most of the heavy solids falling to the bottom of the settling and sedimentation tank, separating suspended solid content from the liquid component of the wastewater. In addition, any surface floating materials can be drained off [24].

#### 2.1.3. Secondary Treatment Process

During the secondary wastewater treatment process, the remaining suspended solids are decomposed and the microbial load is greatly reduced [25]. Typically, this is achieved by bringing the sewage, heterotrophic bacteria and oxygen together in trickling filters or within an activated sludge process, where mixture occurs by mechanical agitation or though mixing by air diffusers. This process utilizes oxygen and bacteria to stabilize the sewage, removing 85–90% of the Suspended Solids (SS) and Biological Oxygen Demand (BOD) [26,27,28]. Table 2 gives an overview of commonly used secondary wastewater treatment processes in South Africa.

#### 2.1.4. Tertiary Treatment Process

Tertiary treatment further removes suspended solids, organic ions and nutrients such as phosphorus and nitrogen from the wastewater prior to release into a receiving watercourse [28]. This treatment process may incorporate procedures such as filtration, phosphorus removal, ammonia stripping and other special treatments which remove specific constituents from the wastewater. Other modes of treatment that may be used include sand filtration, wetlands or other advanced treatment processes. The process of disinfection improves the microbial quality of the wastewater (ideally bringing it to standard) before release into the environment. Some well-known methods of disinfection used in South Africa are: (1) Chemical, e.g., chlorination and ozonation; (2) Physical, e.g., ultraviolet radiation and microfiltration; and (3) Biological, e.g., detention ponds [29].

## 3. Overview of Some South African Wastewater Regulatory Legislation

“The Constitution of South Africa (Act 108, 1996) guarantees everyone the right to an environment that is not harmful to their health or wellbeing and guarantees the right to have the environment protected, for the benefit of present and future generations” [30]. This lays the foundation for a more equitable society through reasonable legislative frameworks. It is the basis of all regulatory procedures/policies that govern wastewater management, from the construction of a wastewater plant, wastewater treatment, to the release of the final effluent [6].

The improvement of supporting legislation or strategies at par with the provision of water services in South Africa is related directly to the primary impact of potable and wastewater on public health [6]. Some of the key legislation related to wastewater management addresses issues such as setting up a treatment plant and release of final effluent. The regulatory legislation has been compiled in Table 3.

## 4. Wastewater Management and Challenges

The paramount aim of wastewater treatment is to enable the disposal of wastewater that is not detrimental to the environment. The implementation of proper wastewater management strategies is not an option but an imperative in order to drive compliance with the set discharge standards. Wastewater management strategies safeguard the sustainable quality of water sources, reducing cost implications involved in drinking water treatment, and deterring or eradicating waterborne diseases [8].

The Department of Water Affairs and other related Water Quality Management units/stakeholders in South Africa, have the responsibility to ensure water quality management [32]. The Department is accountable for the initiation and implementation of water quality policies and documentation, such as the South African Water Quality Guidelines. The guidelines serve as the source for developing materials to advise water users about the physico-chemical, aesthetic and biological properties of water, and consist of the criteria for water quality, Target Water Quality Range (TWQR), and other relevant supporting data [33]. Table 4 shows the indicators for wastewater quality management, highlighting the limit values relevant to discharge of wastewater into a receiving water body [34]. 

A properly monitored and operational wastewater treatment plant is able to release effluent well within standards, eliminating up to 90% of bacterial and viral pathogens [35]. However, despite significant progress that has been made in relation to water quality management, anomalies in the area of wastewater treatment still exist, with there being significant differences between those in affluent and poorer communities [36]. Owing to factors such as the poorly functioning state and inadequate maintenance of some wastewater treatment plants, as well as a lack of facilities to monitor micro-pollutant content of effluent, particularly in poorer communities, the fundamental aim to produce standardized wastewater final effluent remains unrealized in this country. Studies have reported that several wastewater treatment plants still release effluent containing significant amounts of enteric pathogens, in turn resulting in the impairment of the surrounding receiving water bodies and thus posing a serious threat to public health [5,7,8,36]. Enteric pathogens are known to be resilient in wastewater, surviving treatment processes and also being able to develop resistance to chlorine [37].

As mentioned in the introduction, routinely monitored microbial and physicochemical parameters promoted by current water quality guidelines the basis of water quality management and research may not include the organisms causing current illnesses or outbreaks. Studies have already indicated the need to monitor not only the classical pollution indicators but also the highly infectious ones [10]. In South Africa for instance, studies conducted on the occurrence of *Vibrio* pathogens in domestic water and wastewater effluent have made this apparent. A few recent examples are mentioned below.

In a study assessing the prevalence of disease-causing enteric pathogens in rural communities of Nkonkobe, South Africa, 25% of the bacterial isolates obtained from both ground water and surface water samples were confirmed to have toxigenic *Vibrio cholerae* [39]. A previous study on distribution of diarrhoea and microbial quality of domestic water in Khandanama River, Tshikuwi, South Africa, reported indicator microbial counts, which included heterotrophic bacteria, enterococci, total and faecal coliforms [9], exceeding the limits of no risk as indicated by the South African water quality guidelines for domestic use. Further analysis conducted also revealed a high presence of *Shigella*, *Vibrio* and *Salmonella* species in the Khandanama River.

Dungeni et al. [8] further reported on four wastewater treatment plants located in Gauteng Province which had recordings of *Vibrio cholerae* among other related pathogenic bacteria such as *Escherichia coli* and *Salmonella typhimuriam*. The presence of *Vibrio* pathogens in wastewater final effluents has also been found in studies conducted and published by members of the Applied and Environmental Microbiology Research Group (AEMREG) from the University of Fort Hare, South Africa. In a study published in 2010, the *Vibrio* strains *V. parahaemolyticus*, *V. metschnikovii*, *V. fluvialis* and *V. vulnificus* were obtained from wastewater final effluent from a rural community in the Eastern Cape of South Africa [40]. In a comprehensive study conducted in 2012 by Nongogo and Okoh [5], on the occurrence of *Vibrio* pathogens in final effluents from 5 wastewater treatment plants, in the Chris Hani District area in the Eastern Cape Province, South Africa, different species of *Vibrio* were confirmed from 310 isolates, including *V. parahaemoliticus*, *V. vulnificus* and *V. fluvialis*. A study on the presence of *Vibrio* pathogens in final effluents conducted on 14 wastewater treatment plants in the Chris Hani and Amathole district Municipalities, South Africa, reported the presence of *Vibrio* pathogens in most of the wastewater effluent samples in all seasons. Up to 66.8% of the isolates obtained were confirmed to belong to the *Vibrio* genus [11].

## 5. Some *Vibrio* Pathogens in Wastewater Final Effluents in South Africa

The *Vibrio* genus belongs to the family Vibrionaceae, which consists of opportunistic pathogens that affect humans and animals. Common inhabitants of marine coastal ecosystems, it is documented that their changing populations result from changes in seawater temperature in conjunction with warmer temperatures and algal blooms which decline with cooler temperatures. However, their adaptability to adverse conditions is also notably responsible for their presence in other environments such as sewage [8,41,42]. The wide distribution of the Vibrio genus in effluent environments, particularly those associated with domestic sewage has been strongly promoted by their ability to adapt to adverse conditions [43]. Studies have confirmed the association with and resilience of the *Vibrio* genus in wastewater, as they survive and thrive in the harsh conditions of the effluent (despite the various chemical, biological and physical characteristics); this in turn often causes a ripple-like effect of inconsistencies in the receiving water-bodies and the environment as a whole [11,44,45].

*Vibrio cholerae* is responsible for the most common form of *Vibrio* pathology, and results in the gastrointestinal disease cholera through the release of enterotoxins, which causes an efflux of important cell nutrient including sodium and water, leading to diarrhoea and dehydration. *V. cholera* is classified into two serotypes, O1 and nonO1 [46]. The O1 strain is further divided into two biotypes, Classical and ElTor. The biotype ElTor is known to be an emerging causative for *V. cholerae* in humans. Transmitted mostly via the faecal-oral route, cholera has two distinctive features: (1) its propensity to appear and cause sporadic outbreaks/epidemics, and (2) its ability to cause pandemics that spread over many countries over a period of many years [47]. Griffith et al. [48] report that the World Health Organisation receives hundreds of thousands of case reports every year, with these cholera-related epidemics reportedly mostly threatening the developing world. Contaminated wastewater has been associated with the spread of cholera. This is further validated by the high incidence of *V. cholerae* in water samples from various studies, indicating that they are natural inhabitants of aquatic environments [5,11,49].

*Vibrio vulnificus* is known to be extremely virulent and causes three types of infections: (1) severe gastroenteritis from consumption of raw or undercooked seafood; (2) necrotizing wound infections when injured skin is exposed to contaminated water for instance, marine water; and (3) intrusive septicaemia, which is caused when the bacterium invades the blood stream (this is 80 times more likely in immune-compromised individuals such as those with liver disease, such as haemochromatosis [50,51,52,53]. Igbinosa et al. [44] reports that *V. vulnificus* can survive in wastewater effluents even after chlorination, supporting the likelihood of finding it in the receiving waterbodies and surrounding environments after treatment.

*Vibrio fluvialis* causes cholera-like bloody diarrhoea, wound infection and primary septicaemia (prevalent in individuals with a weakened immune system) world-wide [45]. *V. fluvialis* infections are mostly found in areas exposed to increased levels of faecal contaminated food such as contaminated seafood products and water supplies [54]. It may also be transmitted by person-to-person contact [55]. Okoh et al. [40] reports that the association of *V. fluvialis* with low standards of living, inadequate water supply, and poor sanitary conditions have become significant over time.

*Vibrio mimicus* is a *Vibrio* species that mimics *V. cholerae*. It also is known to cause gastroenteritis transmitted from eating raw sea food. In very rare occurrences, in instances where *V. mimicus* is carrying the genes encoding for cholera toxin, it can cause severe watery diarrhoea [55]. *Vibrio alginolyticus* is known for its prevalence in sea water, causing otitis (ear infection), wound infection (skin ulcers), gastroenteritis, blood poisoning (septicaemia), food intoxication and haemorrhaging in humans [56]. *Vibrio parahaemolyticus* has been indicated in the literature to cause acute gastroenteritis, diarrhoea and abdominal pain in individuals who consume unclean seafood (usually raw or undercooked sea food) and, less commonly, wound infections through exposure to sea water.

As mentioned earlier, the presence of *Vibrio* pathogens in wastewater, even those cases documented in predominantly salt water or *Vibrio* pathogens in sea food-related cases, is notable due to their ability to acclimatise quickly to fluctuating environmental conditions. This positions *Vibrio* as an emerging pathogen with implications for public health [57].

## 6. Monitoring of Pathogenic *Vibrio* in Wastewater

The significance of monitoring wastewater effluents has over time become evident due to the presence of microbial pathogens that are considered environmental contaminants or precursors to health-related discrepancies. The process of wastewater treatment may remove most pathogens, but many still remain and are discharged into the effluent, which in turn affects the receiving water bodies and the environment as a whole [58]. In South Africa, the presence of environmental contaminants in wastewater effluents is not unusual, with notable significant differences between effluent in affluent and poorer communities [36]**.**

In conjunction with classical microbiological indicators, the need to monitor outbreak-related pathogens, for example, viral pathogens, protozoa, toxic *E. coli*, highly infectious bacterial pathogens such as *Vibrio* spp., etc., as well as the need to perform direct analysis of specific pathogens of concern is paramount. These pathogens are notably more resilient in the environment with devastating effects. In wastewater for instance, these pathogens are able to withstand wastewater treatment processes, as well as lie dormant or survive for long periods of time. [36].

### 6.1. Conventional Methods

Phenotypic techniques are often used for identifying and enumerating *Vibrio* species. Culture-based techniques may include the pre-enrichment of samples in selective media, plating on to selective media followed by morphological and biochemical characterization [59,60]. For wastewater, for instance, the membrane filter technique is often used. In this technique, a water sample is filtered using a sterile filter with a 0.45 mm pore size, which will help retain bacteria, followed by placing of the filter on a selective medium such as thiosulphate citrate bile salts sucrose (TCBS) agar and appropriate incubation (at 37 °C for 24–48 h), after which typical colonies (green and yellow) will be enumerated on the filter [61]. Table 5 indicates the typical morphology of *Vibrio* pathogens when grown on TCBS agar. A predominant flaw in the membrane filter technique is that it is not able to recover injured bacteria, as a number of chemical and physical factors in the wastewater treatment processes, as well as disinfection, can cause sub-fatal injury to pathogens of interest, resulting in the lack of formation of clear and visible colonies on selective media. This shortcoming is often curbed by a pre-enrichment step using a broth, like alkaline peptone [61].

### 6.2. Molecular Methods

The use of molecular methods for identification and quantification of pathogens, as well as rapid and sensitive characterization of bacteria with unique growth or biochemical requirements, may be seen as a valid alternative approach to the conventional or culture-based methods. Studies have shown that molecular methods are able to detect even the lowest mass of bacteria, and even those that are viable but non-culturable [63,64]. These techniques are also reportedly sensitive towards new emergent strains and indicators, such as *Vibrio* pathogens [58,65,66]. Considering the increasing importance of *Vibrio* spp. in conjunction to the environment and public health as a whole, the molecular method is an acceptable and reliable alternative technique for routine microbial screening and monitoring of environmental and food samples. These methods may also be used in the refinement and evaluation of disease and non-disease causing bacterial strains [58].

The Polymerase Chain Reaction (PCR)-based technique is one of the well-known molecular techniques used to amplify specific DNA sequences; however, PCR-based assays cannot differentiate between live and dead cells. Other regularly used molecular approaches for *Vibrio* species identification are Real-Time PCR, Restriction Fragment Length Polymorphism (RFLP), Microarrays, Multilocus Enzyme Electrophoresis (MLEE), Fluorescence in Situ Hybridization, Multilocus Sequence Typing (MLST), Ribotyping and Amplified Fragment Length Polymorphism (AFLP) [66].

## 7. Implications of Pathogenic Vibrio for Public Health

Enteric pathogens have been documented to cause many of the disease outbreaks that occur worldwide. Pathogenic *Vibrio*, for instance, is becoming a cause for great concern, especially considering the extent to which infections by these organisms are increasing globally. This is accentuated by the constant environmental changes like warmer waters due to climate change. *Vibrio*-related infections are responsible for some of the most deadly and costly food- and water-borne diseases [48].

Due to the severity of disease caused by *Vibrio cholerae*, it has been the focus of recent *Vibrio* species water related research [67]. However, over the last decade the relevance of the more minor *Vibrio* species has become significant. Some studies have reported a few *Vibrio* species of medical importance, which are being described as emerging pathogens that are able to cause mild to severe human diseases [60].

### Brief Epidemiology of Diarrheal/Vibrio Pathogen-Related Health Cases in South Africa

Documented cholera outbreaks in South Africa date back as far as the 1980s, when sporadic outbreaks occurred. The beginning of the worst cholera epidemic, however, was experienced in August 2000, culminating to the deaths of 232, and the infection of about 106,389 people nationwide. The epidemic traversed Kwazulu-Natal, Gauteng, Mpumalanga and the Northern Province. The 2000/2001 epidemic subsided with 3901 reported cases and 45 deaths in Mpumalanga, Eastern Cape and Kwazulu-Natal in 2003. In 2004, 1773 cases of cholera infections and 29 deaths were reported in Mpumalanga’s Nkomazi Region. In the same year, the Eastern Cape had reports of 738 people who were diagnosed with cholera, with four fatalities. Two deaths were also reported in the North West, with 260 cases of infection [68,69,70]. In 2007, 80 diarrhoea-related child deaths occurred in the Eastern Cape, in the Greater Barkly East Area, Ukhahlamba District Municipality [71]. Cholera was confirmed as being endemic in South African water resources by a spokesperson from the South African ministry of health in 2009 [72].

The Limpopo Province is prone to disease outbreaks, especially during rainy seasons, with reports of cholera and malaria being predominant. In 2014, it was reported that at least 45 people were admitted to Voortrekker Hospital after contracting diarrhoea. Nine of those admitted to hospital were in a critical condition. Contaminated water or food was suspected to be the source of the outbreak [73]. 

During the same year, an outbreak of diarrhoea was reported in Fort Beaufort and the surrounding areas, with some deaths and many hospitalizations; residents credited the incident to poor water quality supplied by the municipality [74]. Another outbreak causing infant mortalities was reported in Upington in the same year [75].

There was a contaminated water related scare in Chris Hani District Municipality in the Cradock area in 2015, with thirteen people, including two four-year-olds, treated at the Cradock hospital for gastroenteritis. Although cause of disease was not confirmed, the Health24 resident doctor Dr Heidi van Deventer was of the opinion that the disease could have been caused by either viral or bacterial sources [76].

## 8. Conclusions

Public health discrepancies related directly to water and sanitation in South African are taking centre stage. Emerging outbreak-related pathogens such as *Vibrio* are of importance due to their catastrophic health related implications. It is apparent that there is a need to monitor these pathogens. In the context of wastewater management, the need to perform direct analysis of specific pathogens of concern over and above the routinely monitored classical microbiological indicators is evident. Many of the outbreak-related pathogens present in wastewater do not form part of the routinely monitored indicators. Identifying key techniques/methods that can be used for the detection of *Vibrio* pathogens is needed for good public health. Recommendations have been made, following the detection of pathogenic vibrio species in wastewater treatment plants, to enforce environmental regulations and safeguard the impairment of receiving water bodies. However, proactive strategies should be employed to ensure effective monitoring of pathogenic vibrio species in the plants through efficient management that complies with set guidelines.

## Figures and Tables

**Table 1:** Wastewater-related contaminants.

Contaminants	Impact on Aquatic Environments
Pathogenic Organism	In wastewater they are notably detrimental, responsible for health-related discrepancies.
Suspended Solids (SS)	Suspended solids in untreated wastewater, when accumulated, may lead to the development of sludge deposits. These deposits can notably increase anaerobic conditions in aquatic environments.
Biodegradable organics	Commonly measured as BOD and COD, biodegradable organics consist of proteins, carbohydrates and fats. The discharge of these organics into receiving water bodies (rivers, lakes etc.,) may interfere with biological stabilization, for example, depleting the natural oxygen resources causes septic conditions detrimental to aquatic species.
Priority pollutants	Some organic and inorganic compounds present in wastewater are highly toxic, carcinogenic, mutagenic or teratogenic.
Refractory organics (surfactants, phenols and agricultural pesticides)	These are organics that tend to resist conventional waste-water treatment. Their accumulation in the environment may cause severe problems for the environment for instance environmental poisoning.
Heavy metals (arsenic, lead, mercury, cadmium, chromium, copper, nickel, silver, and zinc)	Usually added by commercial and industrial activities, they are notably the most persistent pollutants in wastewater. The release of high levels of heavy metals into receiving water bodies may cause serious health and environmental complications.
Dissolved inorganics (calcium, sodium and sulphate)	These are often present in domestic waste and must be removed, especially if the wastewater is intended for reuse, e.g., for irrigation purposes. Dissolved inorganics may have a long-term impact on the environment that increases with the continued use of wastewater.

Source: [17].

**Table 2:** Overview of commonly used secondary wastewater treatment processes in South Africa.

Treatment	Description
Trickling filters (bio-filters)	Organic matter is removed from wastewater using trickling or bio-filters. This is an aerobic treatment system that utilizes microorganism populations (bacteria, fungi, algae, and protozoa) attached to a medium forming a biological film approximately 0.1 to 0.2 mm thick to remove organic matter from wastewater. Wastewater passing through the medium with the microorganisms gradually attaches to the rock or plastic surface of the filters forming a film; organic material is degraded by the aerobic microorganisms in the outer part of the slime layer.
Rotating biological contractors	Rotating biological contractors are man-made aerobic attached-growth treatment disk systems, which are attached to shafts mounted over the wastewater to be treated. During treatment the shaft needs to rotate slowly so that the disks are immersed in the wastewater for a short period of time before returning to the air. This ensures the development of biological slime on the disks similar to that of the bio-filter. The developed slime falls back off into the wastewater where it will settle out and be removed or recycled.
Activated sludge processes	The activated sludge process removes organic matter from the wastewater by utilizing the high concentrations of microorganisms (mostly bacteria and some protozoa) present as floc. The floc is kept suspended in the wastewater through agitation. The main processes in the removal of the organic material are adsorption, carbonaceous oxidation, and nitrification. The key components for an activated sludge process are (1) Wastewater passes through a reactor (aeration tank), brought into contact with the present microorganisms; (2) The process transfers oxygen to the microorganisms; (3) The suspension is agitated; (4) The system separates the treated water from the microorganisms; (5) Live microorganisms are put back into the reactor and dead ones are removed.
Sedimentation tanks/Clarifier	The process of secondary sedimentation/clarification is necessary to remove high concentrations of sloughed biomass accumulated from the activated sludge process; separating it from the liquid.

Source: [19,25].

**Table 3:** South African wastewater regulatory legislation.

Acts and Regulatory Legislations	Description
Environment Conservation Act (ECA) (No. 73 of 1989)	In January 1994, the Environmental Conservation Act was adopted to provide for economic growth and social welfare which is environmentally friendly (without negatively influencing, overstraining or irreversibly harming the natural environment and natural resources). A further consequential principle towards the polluter was incorporated in September 1994, stipulating the charges against the polluter for the negative environmental consequences of disposal or discharge actions.
Occupational Health and Safety Act (OHSA) (No. 85 of 1993)	A wastewater plant is required to comply with OHSA in its design and treatment requirements.
National Environmental Management (NEMA) Act (No. 107 of 1998)	NEMA contains internationally accepted principles of sustainability that concur with the South African Constitution (Section 24). Taking these principles into consideration is a legal requirement in all decisions that may affect the environment. Therefore, this is a prerequisite for intergovernmental co-ordination and harmonisation of policies relating to the environment. The Best Practical Environmental Option (BPEO) is defined in NEMA as “the option that provides the most benefit or causes the least damage to the environment as a whole, at a cost acceptable to society, in the long term as well as the short term”.
The Environmental Management Plan (EMP)	This is recognised as the tool within NEMA that provides the assurance that any environmental related project makes suitable provisions for mitigation (Environmental Impact Assessment). An Environmental Impact Assessment (EIA) provides description of methods and procedures for mitigating and monitoring impacts through appropriate objectives. These methods take into consideration the various role players and responsibilities, timescales and cost.
The Water Services Act (No. 108 of 1997)	The Water Service Act designates the role of a Municipality as one of the major role players in Water and Sanitation Management providing an institutional framework for disseminating national norms and standards for providing water services. This simply means the authorisation of a service must be in harmony with funding mechanisms in place and EIA regulations.
The National Water Act (No. 36 of 1998)	This act conceptualises the management of water resources in South Africa based on constitutional rights. It positions the National Government as the custodian of water as a national resource. Provisions for the protection, use, development, conservation, management and control of water resources in the country are affirmed by the act. Setting up a new wastewater treatment plant requires a Water Act license from the Department of Water Affairs (DWA) and a Waste Act license from the Department of Environmental Affairs (DEA).

Source: [6,31].

**Table 4:** South African National Water Act waste discharge standard guidelines.

Variables and Substances	General Standards
Chemical oxygen demand	75 mg/L
Colour, odour or taste	No substance capable of producing the variables listed
Ionised and unionised ammonia (free and saline ammonia)	3 mg/L
Nitrate	15 mg/L
pH	5.5–9.5
Phenol index	0.1 mg/L
Residual chlorine (Cl)	0.25 mg/L
Suspended solids	25 mg/L
Total Aluminium (Al)	-
Total Cyanide (Cn)	0.02 mg/L
Total Arsenic (As)	0.02 mg/L
Total Boron (B)	1 mg/L
Total Cadmium (Cd)	0.005 mg/L
Total Chromium III (CrIII)	-
Total Chromium VI (CrVI)	0.05 mg/L
Total Copper (Cu)	0.01 mg/L
Total Iron (Fe)	0.3 mg/L
Total Lead (Pb)	0.01 mg/L
Total Mercury (Hg)	0.005 mg/L
Total Selenium (Se)	0.02 mg/L
Total Zinc (Zn)	0.1 mg/L
Faecal Coliform	1000 cfu/100 mL

Source: [38].

**Table 5:** Typical morphology of presumptive *Vibrio* pathogens grown on thiosulphate citrate bile salts sucrose (TCBS) agar.

Vibrio Pathogen	Colony Morphology (Colour)
*Vibrio cholerae*	Large yellow colonies
*Vibrio vulnificus*	Yellow-Greenish yellow colonies
*Vibrio fluvialis*	Yellow colonies
*Vibrio mimicus*	Green colonies
*Vibrio parahaemolyticus*	Blue colonies with green centres
*Vibrio alginolyticus*	Large yellow colonies

Source: [62].
